# Prevalence and antimicrobial susceptibility patterns of methicillin-resistant *Staphylococcus aureus* among janitors of Mekelle University, North Ethiopia

**DOI:** 10.1186/s13104-018-3399-1

**Published:** 2018-05-11

**Authors:** Atsebaha Gebrekidan Kahsay, Dawit Gebreegziabher Hagos, Getahun Kahsay Abay, Tadele Araya Mezgebo

**Affiliations:** Department of Medical Microbiology and Immunology, Institute of Biomedical Sciences, College of Health Sciences, Mekelle University, Mekelle, Ethiopia

**Keywords:** Mekelle University, Methicillin resistant *Staphylococcus aureus*, Janitors, Antimicrobial susceptibility tests

## Abstract

**Objective:**

The objective of this study was to determine the prevalence of methicillin-resistant *Staphylococcus aureus* and antimicrobial susceptibility patterns among janitors working at Mekelle University, Tigray, Northern Ethiopia.

**Result:**

The overall prevalence of *S. aureus* and MRSA in the present study were 17.97% (69/384) and 6.25% (24/384) respectively. Although not statistically significant, the prevalence of MRSA among janitors working in the medical area (9.7%, 10/103) was two times higher than the non-medical area (4.9%, 14/281). Janitors who had more service year and who were unable to read and write were found with high isolates of MRSA. Nasal carriage of MRSA among janitors who work in the hospital and who were hospitalized in the last 3 months and those who had exposure to wastes and body fluids were 13 (37.1%) and 10 (38.5%) respectively. Majority of the isolates of *S. aureus* were sensitive to ciprofloxacin (67; 97%), doxycycline (56; 81%), erythromycin (54; 78%), chloramphenicol (50; 72.5%) and cefoxitin (45; 65.2%). Sixty-seven of the 69 (97%) were resistant to penicillin. Of the 69 isolates of *S. aureus*, 22 (31.9%) showed multidrug resistant. Fourteen were resistant to three antimicrobials, 2 were resistant to four antimicrobials, and 7 were resistant to five antimicrobials.

## Introduction

*Staphylococcus aureus*, a Gram-positive spherical-shaped bacterium, predominantly colonizes the skin and nasal mucosa of healthy individuals globally [1]. Approximately 28% of healthy adult individuals have their anterior nares persistently colonized with this bacterium (https://www.ncbi.nlm.nih.gov/pmc/articles/PMC172932) [2]. Person to person transmission of *S. aureus* occurs mostly through contact with fluids from infected skin abrasions [3]. Infections due to *S. aureus* range from simple skin infections to fatal bacteremia and pneumonia [1]. *S. aureus* contributes to one-third of surgical site infections and close to a quarter of ventilator-associated pneumonia [4]. Between 2001 and 2009, the *S. aureus* skin and soft tissue infections associated hospitalization has increased from 39 to 51% in hospitals of USA [5].

With the emergence of methicillin-resistant *S. aureus* (MRSA) first recognized from hospitalized patients in 1960 [4], the treatment of Staphylococcal infections has become more challenging [5]. Until 1990, could only be described as a hospital-acquired infection [6]. Subsequently, MRSA has become a major global public health problem in both hospitals [6–9] and community [10–13] settings globally. Among hospitals and non-medical janitors in Taiwan, 3.6 and 1.3% have MRSA in their nares respectively [14]. In Pakistan, 13.6% of sanitary and 2.1% administrative workers were found to have MRSA in their nares respectively [15]. The nasal carriage of MRSA among 600 randomly selected health workers in Iran was 5.3% [16].

Thirty-four (28.8%) and 15 (12.7%) healthcare workers in Dessie-Ethiopia had their anterior nares colonized with *S. aureus* and MRSA respectively [17]. Eighty-five (59%) and 44 (28.9%) in patients in Jimma-Ethiopia had their anterior nares colonized with *S. aureus* and MRSA respectively [18].

At Mekelle Hospital-Ethiopia, the nasal and hand carriage of MRSA among healthcare workers was 20.3% [19].

In Ethiopia, as to our knowledge, despite its importance, there is no published data that indicates the prevalence of MRSA and antimicrobial susceptibility patterns among janitors of any institution.

Therefore, this research project assessed the prevalence of MRSA and its antimicrobial susceptibility patterns among janitors working in Mekelle University; with a view to providing information to aid the design of appropriate prevention strategies.

## Main text

### Materials and methods

#### Study area and study design

Mekelle University is one of the largest Universities in Ethiopia. It has more than 30 thousand first-degree, second-degree and third-degree annual students in the five campuses [20]. It conducts teaching, research, and community activities. There are 576 janitors working at Mekelle University (155 from one campus in Ayder comprehensive specialized hospital and 421 from other four campuses of the University. A cross-sectional study was conducted from January to May 2016.

### Sample size and sampling technique

A total of 384 janitors (103 from medical and 281 from non-medical) were recruited to participate in the study. The sample size was calculated using single proportion formula (P = 0.5, Zα/2 = 0.196, CI = 95). Simple random sampling technique was used to select 384 study participants. Study participants who had nasal bleeding at the time of data collection were excluded because rolling of swab may aggravate bleeding.

#### Data collection and processing

Trained data collectors were recruited to collect the socio-demographic, clinical and other factors from consenting participants using a standardized questionnaire. The questionnaire could be self-administered (for literate participants) or interview-administer (for participants unable to read and write their mother language).

#### Sample collection, transport, and processing

Using aseptic procedures, trained study assistants took nasal swab samples from both anterior nares of the study participants which were transported to Mekelle University College of Health Sciences department of medical microbiology laboratory within 30 min of collection. Upon arrival, specimens were inoculated on to Mannitol salt agar (HI Media Laboratories, Pvt. Mumbai, India) and incubated at 37 °C for 24 h. After incubation, the presence of *S. aureus* was confirmed by golden yellowish colonies, Gram stain and a biochemical test using slide coagulase test [21, 22].

### Antimicrobial susceptibility tests

Isolates identified as *S. aureus* were subjected to antimicrobial susceptibility tests using the Kirby Bauer disk diffusion method as described by CLSI [23]. Six antimicrobials were used; these were penicillin (10 units), erythromycin (15 μg), chloramphenicol (30 μg), doxycycline (30 μg), ciprofloxacin (5 μg) and cefoxitin (30 μg). Three to five colonies of *S. aureus* were picked from the nutrient agar to make a suspension that was standardized to 0.5 McFarland standards. Using sterile applicator cotton swab, standardized suspensions were swabbed on to the Muller Hinton agar. The antimicrobial discs were placed then on the Muller Hinton agar within 20 min using aseptic technique. The Muller Hinton agar plates were incubated at 35 °C for 18 h. After this, the diameter of the “zone of inhibition” around the antibiotic discs was measured using calipers and reported as sensitive, intermediate and resistance [21–23].

Methicillin resistance *S. aureus* was confirmed by 30 μg cefoxitin disc. A “zone of inhibition” of ≤ 21 mm was considered as methicillin-sensitive and a “zone of inhibition” of > 22 as methicillin-resistant.

#### Quality control

Each lot and shipment of the medium was checked for expiration dates prior to use as part of quality control. The performance of the media and antibiotic discs were evaluated using positive controls (*S. aureus*, ATCC-25923) and negative controls (*Escherichia coli*-ATCC-25922) [23].

### Statistical analysis

Frequency and percentage were used to summarize categorical data. Chi square statistics were used to describe associations between various participants’ characteristics and Staphylococcus nasal carriage. The level of significance was set at α = 0.05 using the two-tailed method. SPSS for Windows Version 20 software was used for the statistical analysis of the collected data.

#### Ethical consideration

Ethical approval was obtained from the Ethical Review Committee of Mekelle University, College of Health Sciences with a proposal number of ERC 0682/2015. Informed consent was collected from each study participants after they understood the aim of the study. As the janitors were outsourced to a private company, permission was collected from the private limited company.

### Results

#### Socio-demographic characteristics

Three hundred and eighty-four participants were recruited. Their mean age was 21 (± 4.97) years and all (100%) were female. The majority (85.2%; 327) were aged 18–24 years. The majority were single (81.8%), had attained a high school education; grade 9–12 (53.1%) and lived with children aged < 15 years (52.6%).

Majority (63%) had served as a janitor for less than 1 year, in a non-medical area (73.1%), did not suffer from allergic rhinitis (95.3%), had not had a skin infection (97.1%), nor a wound infection (94.8%), nor been hospitalized (64.3%) in the 3 months preceding the interview. The majority (73.2%) reported no history of exposure to waste and body fluids (Table 1).

**Table 1:** Prevalence of *Staphylococcus aureus*, MRSA, and MSSA among janitors of Mekelle University, 2016

	Study subjects N = 384, N (%)	Total *S. aureus* isolates N (%), 69 (17.97%)	MRSA^a^, N = 24	MSSA^b^, n = 45	P value
			N (%)	N (%)	
Age					0.708
18–24	327 (85.2)	59 (18)	20 (33.9)	39 (66.1)	
> 24	57 (14.8)	10 (17.5)	4 (40)	6 (60)	
Sex					
Female	384 (100)	69	24	45	–
Male	0 (0)	–	–	–	
Marital status					0.166
Single	314 (81.8)	55 (17.5)	22 (40)	33 (60)	
Married	52 (13.5)	11 (21.2)	2 (18.2)	9 (81.8)	
Divorced	18 (5.7)	3 (16.7)	0	3 (100)	
Educational status					0.038
Unable to read and write	25 (6.5)	8 (32)	5 (62.5)	3 (37.5)	
Junior secondary school	84 (21.9)	17 (20.2)	8 (47.1)	9 (52.9)	
High school	204 (53.4)	33 (16.2)	6 (18.2)	27 (81.8)	
Diploma	71 (18.5)	11 (15.5)	5 (45.5)	6 (54.5)	
Live with children					0.054
Yes	202 (52.6)	28 (13.9)	6 (21.4)	22 (78.6)	
No	182 (47.4)	41 (22.5)	18 (43.9)	23 (56.1)	
Service as a janitor in year					0.023
< 1	242 (63)	43 (17.8)	17 (39.5)	26 (60.5)	
1–2	109 (28.4)	18 (16.5)	2 (11.1)	16 (88.9)	
> 2	33 (8.6)	8 (24.2)	5 (62.5)	3 (37.5)	
Cleaning area					0.286
Medical	103 (26.8)	26 (25.2)	10 (38.5)	16 (61.5)	
None medical	281 (73.2)	43 (15.3)	14 (32.6)	29 (67.4)	
Allergic rhinitis disease					0.168
Yes	18 (4.7)	1 (5.6)	1 (100)	0	
No	366 (95.3)	68 (18.6)	23 (33.8)	45 (66.2)	
Respiratory infection (within last 3 months)					0.168
Yes	11 (2.9)	1 (9.1)	1 (100)	0	
No	373 (97.1)	68 (18.2)	23 (33.8)	45 (66.2)	
Skin infection (within last 3 months)					0.005
Yes	11 (2.9)	4 (36.4)	4 (100)	0	
No	373 (97.1)	65 (17.4)	20 (30.8)	45 (69.2)	
Wound present (within last 3 months)					0.938
Yes	20 (5.2)	6 (30)	2 (33.3)	4 (66.7)	
No	364 (94.8)	63 (17.3)	22 (34.9)	41 (65.1)	
Hospital stay and hospitalization (within 3 months)					0.676
Yes	137 (35.7)	35 (25.5)	13 (37.1)	22 (62.9)	
No	247 (64.3)	34 (13.8)	11 (32.4)	23 (67.6)	
Exposure to waste and fluids					0.618
Yes	103 (26.8)	26 (25.2)	10 (38.5)	16 (61.5)	
No	281 (73.2)	43 (15.3)	14 (32.6)	29 (67.4)	

N total isolates of MRSA, n total number of MSSA isolates

^a^Methicillin resistance *S. aureus*

^b^Methicillin sensitive *S. aureus*

#### Isolation of *S. aureus* from participant’s samples

Samples were collected from the anterior nares of all participants; *S. aureus* was cultured from 69 (17.97%) of the samples. Of the 69 isolates, 25.2% (26/103) were recovered from janitors that worked in medical areas and 15.3% (43/81) from those that worked in non-medical areas of the University (Table 1).

#### Prevalence of MRSA among janitors working at Mekelle University

Among participants with positive isolates (n = 69), 24 (34.8%) had methicillin resistant *S. aureus*. Ten of the 26 isolates (38.5%) were among janitors working in medical areas where as 14/43 (32.6%) was working in non-medical areas. The overall prevalence of MRSA was 6.25% (24/384) (Table 1).

### Antimicrobial susceptibility patterns of *S. aureus* isolates

All the 69 *S. aureus* isolates were subjected to antimicrobial susceptibility tests against seven antimicrobials. Majority of the isolates of *S. aureus* were sensitive to ciprofloxacin (67; 97%), doxycycline (56; 81%), erythromycin (54; 78%), chloramphenicol (50; 72.5%) and cefoxitin (45; 65.2%). Sixty-seven of the 69 (97%) were resistant to penicillin. About one-third 24 (34.8%) of the isolates were resistant to cefoxitin. Of the 45 methicillin susceptible *S. aureus*, 2 (4.4%), 5 (11.1%), 6 (13.3%), 6 (13.3%) and 43 (95.6%) were resistant to ciprofloxacin, doxycycline, erythromycin, chloramphenicol, and penicillin respectively (Table 2).

**Table 2:** Antimicrobial susceptibility patterns of *S. aureus* isolates in Mekelle University, 2016

Antimicrobial drugs	*S. aureus*		Total	P value
	MRSA, N = 24	MSSA, n = 45	N (%)	
	N (%)	N (%)		
Ciprofloxacin				
Susceptible	24 (100)	43 (95.6)	67 (97.1)	0.295
Resistance	–	2 (4.4)	2 (2.9)	
Doxycycline				
Susceptible	16 (66.7)	40 (88.9)	56 (81.2)	0.025
Resistance	8 (33.3)	5 (11.1)	13 (18.8)	
Erythromycin				
Susceptible	15 (62.5)	39 (86.7)	54 (78.3)	0.020
Resistance	9 (37.5)	6 (13.3)	15 (21.7)	
Chloramphenicol				
Susceptible	11 (45.8)	39 (86.7)	50 (72.5)	0.000
Resistance	13 (54.2)	6 (13.3)	19 (27.5)	
Cefoxitin				
Susceptible	–	45 (100)	45 (65.2)	
Resistance	24 (100)	–	24 (34.8)	
Penicillin				
Susceptible	–	2 (4.4)	2 (2.9)	0.295
Resistance	24 (100)	43 (95.6)	67 (97.1)	

MRSA, methicillin resistance *Staphylococcus aureus*; MSSA, methicillin sensitive *Staphylococcus aureus*

### Multidrug resistance (MDR) isolates of *S. aureus*

Of the 69 isolates of *S. aureus*, 22 (31.9%) isolates showed MDR. Fourteen were resistant to three antimicrobials, 2 were resistant to four antimicrobials, and 7 were resistant to five antimicrobials (Table 3).

**Table 3:** Multidrug-resistance isolates of *S. aureus* among janitors of Mekelle University, 2016

Number of antimicrobial resistance	*Staphylococcus aureus* isolates (n = 69)	
	MDR patterns (n = 22, 31.9%)^a^	Number of isolates (%)
Three	CIP, Pen, C	1 (1.4)
	Fox, Pen, C	5 (17.2)
	E, Doxy, Pen	1 (1.4)
	Fox, E, Pen	3 (4.3)
	Fox, Doxy, Pen	1 (1.4)
	Doxy, Pen, C	3 (4.3)
Four	E, Doxy, Pen, C	1 (1.4)
	Fox, Doxy, Pen, C	1 (1.4)
Five	Fox, E, Doxy, Pen, C	6 (21.4)
	Fox, E, Doxy, CIP, C	1 (1.4)

^a^Fox, cefoxitin (30 μg); C, chloramphenicol (30 μg); Ery, erythromycin (15 μg); Pen, penicillin (10 unit); CIP, ciprofloxacin (1 μg); Dox, doxycycline (30 μg)

#### Factors associated with MRSA colonization of the anterior nares among janitors at Mekelle University

A significantly higher proportion of participants with *S. aureus* colonization was found among participants who were unable to read or write compared to those who had attained junior secondary, high school and diploma level education (62.5% vs. 47.1, 18.2, and 45.5% respectively); among participants that had served as janitors for more than 2 years compared to those who had served for less than 1 year and for between one and 2 years (62.5% vs. 39.5% and 11.1% respectively) (Table 1).

*Staphylococcus aureus* was isolated from the nasal swab of one study participants who had allergic rhinitis and was resistant to cefoxitin/methicillin. Nasal carriages of MRSA among janitors who work in the hospital and who were hospitalized in the last 3 months and those who had exposure to wastes and body fluids were 13 (37.1%) and 10 (38.5%) respectively (Table 1).

### Discussion

The overall prevalence of *S. aureus* and MRSA in this study were 69 (17.97%) and 24 (6.25%). This is lower than the study carried out among hospital personals in Pakistan [15] and school children and prisoners in Jimma-Ethiopia [3], but similar to the study revealed from healthcare workers in Iran [16].

*Staphylococcus aureus* was found to be colonized in 26 (25.2%) janitors working in medical and 43 (16%) in non-medical area, whereas MRSA revealed from 10 (9.7%) in medical and 14 (4.9%) non-medical area. This is higher than the study carried out in Taiwan [14]. This may be due to difference in personal hygiene.

Above 85% of the janitors working in the university were youth (18–24 years). The other below 15% was above 24 years and was found to be with high carriage of MRSA 4 (40%). This is comparable with the study among prisoners in Jimma-Ethiopia [3]. Above 70% of the janitor were high school and diploma but high prevalence of MRSA 5 (62.5%, P = 0.038) were revealed among janitors who were unable to read and write in which transmission may be high. This might be due to lack of awareness in using safety measures available in the medical area during work (exposure to wastes) and may have hand nasal contact that can lead to increase the MRSA. About 62% of the participants were served below 1 year as a janitor, however, MRSA carriage was raised among the janitors who served above 2 years 5 (62.5%, P = 0.023).

None of the participants were presented with underlying diseases except eighteen participants with allergic rhinitis and one of whom having allergic rhinitis had MRSA which is not statistically significant. This is different from a study conducted in Taiwan [14]. Janitors were also requested to respond to the occurrence of respiratory infection, skin infection and present of wound, hospital stay and hospitalization in the last 3 months. The carriage rate of MRSA among the janitors having respiratory, skin infection and present with wound were 1 (100%), 4 (100%) and 2 (33.3%) respectively. This is unlike to the study reported from Taiwan [14].

MRSA isolates were 100% resistant to penicillin and cefoxitin which is similar to the study reported from Jimma [3]. 24 (100%), 16 (66.7%), 15 (62.5%), and 11 (45.8%) isolates of MRSA were sensitive to ciprofloxacin, erythromycin, doxycycline, and chloramphenicol respectively.

Six of the isolates of MRSA (8.6%) were resistant to five antimicrobial drugs and the resistant patterns were cefoxitin/erythromycin/doxycycline/penicillin/chloramphenicol.

### Conclusion

Methicillin-resistant *S. aureus* was isolated from both medical and non-medical area. Even though there was no statistically significant difference, the proportion of MRSA isolated from the medical area was two times higher than the non-medical area. Participants who were unable to write and read and those who have at most service year were presented to acquire more MRSA and have the statistically significant difference.

More than 30% of confirmed MRSA isolates were resistant to most of the antimicrobial drugs tested in the present study; however, no resistance was recorded from ciprofloxacin.

Janitors who were unable to write and, having more services and serving in the hospital should get awareness on the impact of MRSA and its transmission especially in relation to the hospital patients during cleaning of in-patient rooms.

## Limitations

This study limited to conduct the MRSA gen as well as some additional antimicrobial agents.

## Abbreviations

*S. aureus*: *Staphylococcus aureus*
MRSA: methicillin resistance *Staphylococcus aureus*
SPSS: Statistical Package for Social Sciences
MDR: multidrug resistance
ERC: Ethical Review Committee
USA: United States of America
CI: confidence interval
ATCC: American type culture collection
CLSI: Clinical Laboratory Standards Institute
